# Translocation of elements and fractionation of Mg, Cu, Zn, and Cd stable isotopes in a penny bun mushroom (*Boletus edulis*) from western Czech Republic

**DOI:** 10.1007/s11356-023-25753-8

**Published:** 2023-02-11

**Authors:** Alexandre V. Andronikov, Irina E. Andronikova, Eva Martinkova, Ondrej Sebek, Marketa Stepanova

**Affiliations:** Division of Geochemistry and Laboratories, Czech Geological Survey, Geologicka 6, 15200 Prague, Czech Republic

**Keywords:** Trace elements, Non-traditional stable isotopes, Translocation, Mushroom, Fruiting body, Soil

## Abstract

*Boletus edulis* mushroom behaved as an accumulating biosystem with respect to Ag, Rb, Zn, and K. The mushroom was not an efficient accumulator of toxic As, Pb, and Cr, but Se and Cd displayed much higher concentrations in the mushroom than in the substrate samples. Other elements were bioexclusive. Different elements had different within-mushroom mobilities. The highest mobilities were displayed by Zn and Ag, and the lowest by Ti. The mushroom’s fruiting body preferentially took up lighter Mg, Cu, and Cd isotopes (Δ^26^Mg_FB-soil_ =  −0.75‰; Δ^65^Cu_FB-soil_ =  −0.96‰; Δ^114^Cd_FB-soil_ =  −0.63‰), and the heavier ^66^Zn isotope (Δ^66^Zn_FB-soil_ = 0.92‰). Positive within-mushroom Zn isotope fractionation resulted in accumulation of the heavier ^66^Zn (Δ^66^Zn_cap-stipe_ = 0.12‰) in the mushroom’s upper parts. Cadmium displayed virtually no within-mushroom isotope fractionation. Different parts of the fruiting body fractionated Mg and Cu isotopes differently. The middle part of the stipe (3–6 cm) was strongly depleted in the heavier ^26^ Mg with respect to the 0–3 cm (Δ^26^Mg_stipe(3–6)-stipe(0–3)_ =  −0.73‰) and 6–9 cm (Δ^26^Mg_stipe(6–9)-stipe(3–6)_ = 0.28‰) sections. The same stipe part was strongly enriched in the heavier ^65^Cu with respect to the 0–3 cm (Δ^65^Cu_stipe(3–6)-stipe(0–3)_ = 0.63‰) and 6–9 cm (Δ^65^Cu_stipe(6–9)-stipe(3–6)_ =  −0.42‰) sections. An overall tendency for the upper mushroom’s parts to accumulate heavier isotopes was noted for Mg (Δ^26^Mg_cap-stipe_ = 0.20‰), Zn (Δ^66^Zn_cap-stipe_ = 0.12‰), and Cd (Δ^114^Cd_cap-stipe_ = 0.04‰), whereas Cu showed the opposite trend (Δ^65^Cu_cap-stipe_ =  −0.08‰).

## Introduction

Biogeochemical cycling in natural environment involves both essential and non-essential elements (Kaspari and Powers 2016; Filipiak and Filipiak 2022). Fungi which are located on the lithosphere to biosphere interface are an important mean of transfer of such elements. Mushrooms concentrate nutrients and minerals from the substrate (organic matter, soil, rocks) through the long aggregated and branching threads named hyphae (Burford et al. 2003; Martino et al. 2003; Fomina et al. 2004; Gadd 2007). Although multiple research has been carried out on trace elements (mainly heavy metals) in mushrooms as possible markers of environmental pollution (Kalac and Svoboda 2000; Kalac 2009 and references therein), there is only a limited number of isotopic study of transition metals in biological systems (potentially important in biogeochemical studies) (e.g., Weiss et al. 2005; Moynier et al. 2009; Ryan et al. 2013; Fahad et al. 2016; Uhlig et al. 2017; Kříbek et al. 2020; Borovicka et al. 2022; Wiggenhauser et al. 2022).

Isotopes as a group represent atoms with the same number of protons and electrons but with a different number of neutrons; stable isotopes are non-radioactive form of atoms (Kendall and Doctor 2003; Teng et al. 2017). During chemical reactions or physical processes, small changes in relative isotope abundances occur, which changes are called isotope fractionation. Isotope fractionation is usually mass-dependent, induced by differences of masses of distinct isotopes (Teng et al. 2017). Isotopes of light elements such as hydrogen, carbon, oxygen, nitrogen, and sulfur (traditional stable isotopes) are long since used in biogeochemical cycling studies whereas the use of non-traditional stable isotopes is very limited in such studies. Presently, non-traditional stable isotopes of Li, Mg, Ca, and Fe are the most used for biogeochemical cycling studies. Stable isotopes of other metals (Sr, Cu, Zn, Cr, Ba, Mo, Hg, Cd, and Ni) are not so widely used yet (Teng et al. 2017; Wiggenhauser et al. 2022 and references therein). Magnesium, Cu, Zn, and Cd isotope systems emerge to be very prospective. Magneisum is a macroelement required in millimolar concentrations for mushrooms successful growth and existence whereas trace elements such as Cu and Zn are generally required in the micromolar range (Gadd 2017). Cadmium and other toxic elements significantly affect plants and fungal growth when are present in high concentrations (Walker and White 2018; Kaspari 2021).

Some non-traditional stable isotopes (Mg, Ca, Cu, Zn) have shown fractionation within vascular plants (e.g., Weiss et al. 2005; Moynier et al. 2009; Bolou-Bi et al. 2010, 2012; Weinstein et al. 2011; Ryan et al. 2013; Kříbek et al. 2020; Wiggenhauser et al. 2022). However, to the best of our knowledge, only a limited number of non-traditional stable isotope studies was conducted up to date for fungi (Fahad et al. 2016; Pokharel et al. 2017, 2018; Andronikov et al. 2022; Borovicka et al. 2022).

In the frame of our study of mushrooms in the Czech Republic, we figured out that non-traditional stable isotopes (Cu, Zn, and Mg) fractionate both on the substrate-to-mushroom interface and within the mushroom’s fruiting body (Andronikov et al. 2022). Additionally, Borovicka et al. (2022) showed that Cd isotopes may insignificantly fractionate during within-mushroom translocation. Magnesium, Cu and Zn are bioessential elements necessary for most living organisms, whereas Cd is a very toxic and carcinogenic metal which is among the most effectively accumulating elements (e.g., Kaspari and Powers 2016; Gadd 2017; Nordberg et al. 2018; Borovicka et al. 2022; Filipiak and Filipiak 2022). We conducted the detailed study of trace element distributions and isotope fractionation of Mg, Cu, Zn, and Cd on the growing substrate to mushroom interface, and within the mushroom’s fruiting body (penny bun mushroom; *Boletus edulis*). *Boletus edulis* is one of the most wide-spread mushrooms in the Czech Republic (Antonin et al. 2019; Knauerova et al. 2020). The conducted studies if extended may offer a new perspective for understanding possible chemical processes in natural environment.

## Materials and methods

**Fig. 1:**
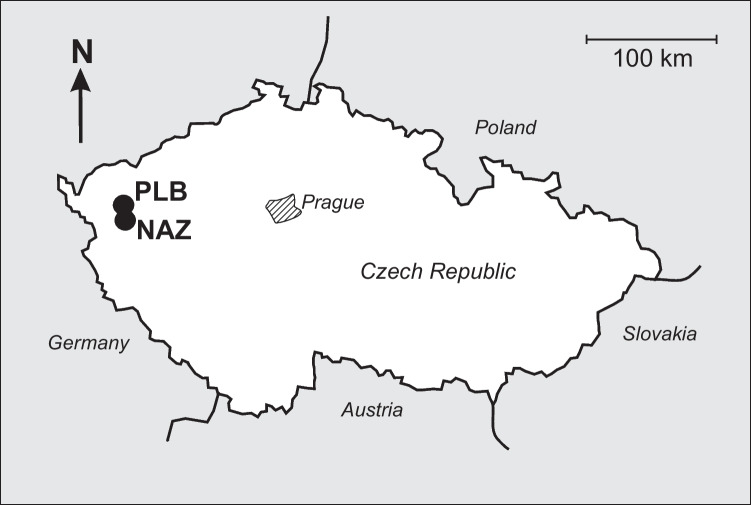
Schematic map showing location of the studied site Pluhov Bor (PLB). NAZ (Na Zelenem), an amphibolite-based catchment where a sample of *Xerocomus subtomentosus* was collected (Andronikov et al. 2022). See text for details

Samples of the fully grown *Boletus edulis* and substrate (soil) were collected in mid-August 2021 in a small forested catchment Pluhuv Bor (0.22 km^2^) located approximately 120 km west of Prague (the capital of the Czech Republic) (Fig. 1). The catchment is underlain by serpentinite bedrock with the soil mostly represented by cambisols, stagnosol and gleysol. Main characteristics of the catchment are given in the Table 1. Two types of substrate samples were collected: (i) from directly under the mushroom down to the depth of 10 cm (mushroom-influenced soil) following sampling strategy described in Busuioc et al. (2011), Falandysz et al. (2017), and Durdic et al. (2021), and (ii) 1.5 m away from the mushroom down to the same depth (further on, we call such samples mushroom- “free” although some mycelium can still be present here). The surface soil layer containing fresh litter, was removed for both types of the substrate. A collected mushroom, after being cleaned of any visible vegetation and soil substrate debris, was separated into sub-samples — the stipe (a half of the stipe cut along the long axis), the cap, and the sporophore. The second half of the stipe was cut into three pieces: 0–3 cm from the bottom, 3–6 cm from the bottom, and 6–9 cm from the bottom. Thereafter, all mushroom sub-samples were air-dried for several days and then at 65 °C to constant weight. Dried fungal sub-samples (500 mg) were pulverized in an agate mortar and put into a pressure resistant, analytical quality pro-digestive polytetrafluoroethylene (PTFE) vessel. The fungal materials were pre-digested for 24 h with concentrated nitric acid (67–69%; Ultrapure, Romil; 7 mL) at the room temperature and further digested under pressure in a MARS 6 (CEM Corp. Matthews, NC, USA) automatic microwave digestion system. The HNO_3_-based digest, after being treated with concentrated HCl and H_2_O_2_, was diluted to 0.3N HNO_3_ using Milli-Q water for further instrumental analysis.

**Table 1:** Study site characteristics

Pluhuv Bor	
Acronym	PLB
Location/Coordinates	Slavkov Forest / 50°04’N 12°46 ‘E
Area (km^2^)	0.22
Elevation (m a.s.l.)	690–804
Bedrock	Serpentinite
Soil types	Cambisols, Stagnosol, Gleysol
Average soil thickness (cm)	100
Vegetation	Norway spruce (*Picea abies*) > 71%; Scotts pine (*Pinus sylvestris*) 10%; Forest clearings 19%
Average annual precipitation (mm)	806
Mean annual air temperature (°C)	+ 6.0

The data are from Novak et al. (2017)

Substrate samples were air dried, sieved to < 2 mm fraction, and then pulverized with the use of an agate mortar. Since the substrate samples contained organic material, they were ashed in platinum dishes at 550 °C before further processing. Substrate samples were finally quantitatively dissolved by a HF–HClO_4_ acid digestion followed by a Na_2_CO_3_–Li_2_B_4_O_7_ oxidative alkaline fusion (Strnad et al. 2005). The samples were then diluted to 50 mL using 0.3N HNO_3_. All sample solutions were filtered through an acid-cleaned syringe filter with a pore size of 0.45 μm before further processing.

### Chemical and isotope analyses

Chemical compositions of the mushroom and substrate samples were determined with the use of the Agilent Technologies 5110 inductively coupled plasma-optical emission spectrometer (ICP-OES) at the Czech Geological Survey. Samples in 0.3N HNO_3_ were introduced into the plasma with a standard quartz cyclonic spray chamber, a concentric glass nebulizer (Meinhard) and an Agilent Technologies SPS-4 autosampler. Operating conditions were as follows: RF power 1200 W, plasma Ar flow 12 L min^−1^, auxiliary gas flow 1.0 L min^−1^, and nebulizer gas flow 0.7 L min^−1^. Wavelengths for individual elements were selected according to the manufacturer’s application sheets. The ICP-OES was operated in the Synchronous Vertical Dual View (SVDV) configuration. All analyses were controlled using blanks and triplicate measurements. The following 25 elements were measured for concentrations in all mushroom and substrate samples: Ag, Al, As, Ba, Ca, Cd, Cr, Cu, Fe, K, Li, Mg, Mn, Na, Nb, Ni, P, Pb, Rb, S, Se, Sn, Sr, Ti, and Zn. Among those, 13 are essential elements for life (Kaspari and Powers 2016).

Sample preparation and isotopic analyses were conducted at the Czech Geological Survey. Sample aliquots containing 20 µg of Mg, 2 µg of Cu and Zn each, and 0.5 µg of Cd were subjected to elution chromatography before the analytical work. Analyses of isotopic compositions of Mg, Cu, Zn, and Cd were conducted using a Neptune (ThermoFisher) multi collector inductively coupled plasma-mass spectrometer (MC-ICP-MS) equipped with an array of nine Faraday detectors. A detailed description of sample preparation and analytical techniques is given in Brenot et al. (2008), Voldrichova et al. (2014), Chrastny et al. (2015), Martinkova et al. (2016), Novak et al. (2016), Pokharel et al. (2017), and Uhlig et al. (2017). The isotopic composition is expressed in the per mil ($\updelta$) notation as per mil (‰) relative to the element’s (E) isotope standard reference material (SRM) as follows:

$${\updelta }^{\mathrm{x}}\mathrm{E(permil) }=\left[\left({ }^{\mathrm{x}}\mathrm{E}/{ }^{\mathrm{y}}{\mathrm{E}}_{\mathrm{sample}}\right)/\left({ }^{\mathrm{x}}\mathrm{E}/{ }^{\mathrm{y}}\mathrm{ESRM}\right)-1\right]\mathrm{x}1000$$

where the “x” and”y” are the heavier and lighter isotopes of the element E, and ESRM is characteristics of the element E in the NIST SRM.

We used the DSM-3 standard for Mg (Galy et al. 2003), ERM-AE633 standard for Cu (Moeller et al. 2012), NIST SRM 683 standard for Zn (Chen et al. 2016), and NIST SRM 3108 standard for Cd (Liu et al. 2020) analyses. Quality control was conducted by replicate analyses of the NIST SRM 1515 (apple leaves), NIST SRM 2709a (soil), NIST SRM 2711a (soil) and the USGS standard BHVO-2 (Hawaiian basalt). The measured isotopic compositions of the standards were within the range of the values reported by Makishima and Nakamura (2013), Sossi et al. (2015), Shalev et al. (2018) and Liu et al. (2020). Procedural blanks were < 15 ng for Mg, < 10 ng for Cu, < 14 ng for Zn, and < 0.2 ng for Cd. These amounts are negligible compared to analyzed material amounts, and no corrections were needed.

A number of authors (e.g., Moynier et al. 2009; Busuioc et al. 2011; Ryan et al. 2013) proposed to use bioaccumulation (BF), translocation (TF) and isotope fractionation ($\Delta$) factors to estimate chemical and isotopic relations between the substrate and the plant (in our case, the mushroom), and within-plant (mushroom) chemical and isotopic relations.

The BF represents intensity of the certain element’s uptake by the mushroom’s fruiting body. The BF is expressed by the Eq.:

$$\mathrm{BF}={\mathrm{C}}_{\mathrm{M}}/{\mathrm{C}}_{\mathrm{S}}$$

where C_M_ is the certain element concentration in the mushroom/its part, and C_S_ is the certain element’s concentration in the substrate. A mushroom is considered to be a bioaccumulating system if the BF > 1 otherwise it is a bioexclusive system.

The TF (translocation is the movement of substances from one place to another through the fungi’s hyphae) is a ratio of the certain element concentration in different mushroom’s parts. The TF is calculated according to the Eq.:

$$\mathrm{TF}={\mathrm{C}}_{\mathrm{upper}}/{\mathrm{C}}_{\mathrm{lower}}$$

where C_upper_ is the concentration of the certain element in the upper part of the mushroom’s fruting body, and C_lower_ is the element concentration in the lower part of the mushroom’s fruiting body. The TF > 1 suggests the high mobility of the element, whereas the TF < 1 suggests the element’s low mobility.

Isotope fractionation factor can serve as the measure of the degree of preferential uptake of the heavy (or light) isotope by the considered mushroom’s part. The Δ is a difference between isotopic composition of the upper part of the mushroom and its lower part (or the substrate). The Δ is expressed by the Eq.:

$$\Delta ={\mathrm{IR}}_{\mathrm{R}}-{\mathrm{IR}}_{\mathrm{S}}$$

where IR_R_ is the isotopic ratio of the mushroom (or its certain part), and IR_S_ is the isotopic ratio of the “source” (substrate or the mushroom’s certain part). The isotopic ratios are expressed as δ notation as per mil (‰) relative to the corresponding isotope SRM.

## Results

The studied mushroom sample contained macro- and trace elements in amounts which were mostly within the ranges reported by for example Kalac and Svoboda (2000), Svoboda and Chrastny (2008), Kalac (2009), Mallikarjuna et al. (2013), and Kojta et al. (2016) for different mushroom types. Different parts of the mushroom’s fruiting body contained 5.0–26 mg kg^−1^ Cu, 29–204 mg kg^−1^ Zn, 0.14–0.40 mg kg^−1^ Cr, 422–917 mg kg^−1^ Mg, 0.28–1.9 mg kg^−1^ Ag, 2.5–7.3 mg kg^−1^ Al, 0.85–1.5 mg kg^−1^ As, 0.12–0.35 mg kg^−1^ Ba, 0.69–3.8 mg kg^−1^ Cd, 7.2–17 mg kg^−1^ Se, 55–119 mg kg^−1^ Ca, 7.7–23 mg kg^−1^ Fe, 11,343–19,195 mg kg^−1^ K, 9246–11,147 mg kg^−1^ S, 0.01–0.13 mg kg^−1^ Li, 3.9–6.7 mg kg^−1^ Mn, 517–1501 mg kg^−1^ Na, 0.83–1.4 mg kg^−1^ Ni, 0.29–0.48 mg kg^−1^ Pb, 0.03–0.19 mg kg^−1^ Nb, 2372–8550 mg kg^−1^ P, 186–408 mg kg^−1^ Rb, 0.07–0.21 mg kg^−1^ Ti, 0.20–0.43 mg kg^−1^ Sr, and 0.41–0.60 mg kg^−1^ Sn.

The bulk stipe of the studied *B. edulis* sample could be considered as an accumulator of elements such as Ag (BF = 3.1), Rb (BF = 1.5), Cd (BF = 1.3), Zn (BF = 1.2), and K (BF = 1.1). Additionally, Se displayed much higher concentrations in the bulk stipe (7.5 mg kg^−1^) than in the substrate (< 0.1 mg kg^−1^, i.e., below the detection limit). However, since we do not know exact concentration of the element in the substrate, we did not calculate the BF for Se, but by all means the BF should be high. Elements such as P and S (BF = 13–14), which are among the main nutrients required for mushroom growth (Gadd 2017; Filipiak and Filipiak 2022), expectedly showed very high BFs (13–14). The rest of the analyzed elements was bioexclusive (BF from < 1 to <  < 1) suggesting that at least in the late summer, a fully-grown *B. edulis* mushroom was not an efficient accumulator of elements.

The BF calculated for the lowermost part of the stipe (0–3 cm) displayed a different picture. Only P and S displayed similarly high BF as in the case of the bulk stipe (11–16). Elements such as Ag (BF = 1.5), Rb (BF = 1.3) and K (BF = 1.0) remaining bioaccumulating though accumulated not as readily as in the bulk stipe (BF = 6.0, 2.6, and 1.5, respectively). Cadmium (BF = 0.9) and Zn (BF = 0.6) even behaved as bioexclusive elements (although Zn is one of the bioessential elements) in the lowermost part of the mushroom’s stipe. Selenium behaved similar as in the case of the bulk stipe (7.5 vs. 7.2 mg kg^−1^, respectively).

The distribution and mobility of elements within the different parts of the mushroom’s fruiting body were characterized by the TF values varying from 0.3 for Ti to 2.6 for Ag but for most elements, TFs varied between 1 and 2 (Table 3). The highest TF_mean_ (TF calculated as a mean of all elements’ TF values) was between the lowermost part of the mushroom stipe (0–3 cm) and its middle (3–6 cm) part (TF_mean_ = 1.25). The lowest TF_mean_ (1.11) was between the uppermost (6–9 cm) stipe part and the cap, i.e., the compositions of the 6–9 cm stipe section and the cap were similar. Sporophore accumulated the highest amounts of Ag (1.85 mg kg^−1^; TF_spor/cap_ = 1.94), Cd (3.82 mg kg^−1^; TF_spor/cap_ = 2.29), Cr (0.40 mg kg^−1^; TF_spor/cap_ = 1.59), Cu (23.8 mg kg^−1^; TF_spor/cap_ = 1.95), Fe (23.3 mg kg^−1^; TF_spor/cap_ = 1.41), Mg (917 mg kg^−1^; TF_spor/cap_ = 1.42), Ni (1.43 mg kg^−1^; TF_spor/cap_ = 1.42), P (8550 mg kg^−1^; TF_spor/cap_ = 1.92), Rb (480 mg kg^−1^; TF_spor/cap_ = 1.22), Se (16.9 mg kg^−1^; TF_spor/cap_ = 1.66), W (1.79 mg kg^−1^; TF_spor/cap_ = 1.75), and Zn (204 mg kg^−1^; TF_spor/cap_ = 1.96) among all analyzed mushroom’s individual parts. Overall, the sporophore tended to accumulate more elements than the cap (mean TF_spor/cap_ = 1.81). On the other hand, elements such as Ba (0.35 mg kg^−1^; TF_3-6/0–3_ = 0.78), Ca (119 mg kg^−1^; TF_3-6/0–3_ = 0.77), Li (0.13 mg kg^−1^; TF_3-6/0–3_ = 0.42), Na (1501 mg kg^−1^; TF_3-6/0–3_ = 0.89), and Sr (0.43 mg kg^−1^; TF_3-6/0–3_ = 0.77) tended to accumulate in the lowermost part of the stipe (0–3 cm) showing therefore low mobilities. All these elements (except for Ca and Na) were represented in the mushroom fruiting body in very low amounts and displayed low BFs (Tables 2 and 3).

**Table 2:** Chemical (mg kg^−1^) and isotopic compositions of the studied mushroom and substrate samples

	Mushroom				
	Stipe 0–3 cm	Stipe 3–6 cm	Stipe 6–9 cm	Stipe Bulk	Cap
Ag	0.28	0.71	0.98	0.60	0.95
Al	2.53	3.57	4.92	3.21	7.33
As	1.31	0.85	1.53	1.03	1.11
Ba	0.35	0.27	0.22	0.20	0.17
Ca	119	91.3	61.5	73.6	69.9
Cd	0.69	1.20	1.40	1.00	1.67
Cr	0.14	0.22	0.26	0.17	0.25
Cu	5.04	8.01	11.37	7.38	12.19
Fe	7.72	11.85	17.63	11.67	16.48
K	11343	14456	17152	12171	19195
Li	0.13	0.06	0.04	0.07	0.02
Mg	422	524	605	448	644
Mn	5.73	5.60	4.97	4.90	6.72
Na	1501	1338	991	1075	517
Nb	0.19	0.07	0.17	0.10	0.07
Ni	0.91	0.95	1.11	0.83	1.01
P	2372	3514	4441	3101	4458
Pb	0.33	0.48	0.33	0.36	0.29
Rb	186	234	300	212	387
S	11147	10400	9549	9246	9508
Se	7.22	8.69	9.22	7.49	10.20
Sn	0.41	0.43	0.41	0.47	0.60
Sr	0.43	0.34	0.22	0.28	0.25
Ti	0.09	0.09	0.13	0.08	0.21
Zn	28.9	67.8	104	61.4	104
δ^26^ Mg ± 2SE	−0.31 ± 0.03	−1.04 ± 0.04	−0.76 ± 0.03	−0.64 ± 0.04	−0.44 ± 0.03
δ^65^Cu ± 2SE	−0.33 ± 0.01	0.30 ± 0.01	−0.12 ± 0.02	−0.05 ± 0.01	−0.12 ± 0.01
δ^66^Zn ± 2SE	0.69 ± 0.03	0.79 ± 0.03	0.82 ± 0.02	0.76 ± 0.02	0.88 ± 0.01
δ^114^Cd ± 2SE	−0.10 ± 0.01	−0.15 ± 0.01	−0.11 ± 0.01	−0.14 ± 0.03	−0.10 ± 0.02

	Mushroom		Soil	
		Fruiting body mean	Mushroom-bearing	Mushroom- “free”
	Sporophore			
Ag	1.85	1.14	0.19	0.29
Al	3.33	4.43	48952	48125
As	1.36	1.17	14.2	14.0
Ba	0.12	0.16	242	233
Ca	55.3	66.2	19,733	19,352
Cd	3.82	2.18	0.77	0.69
Cr	0.40	0.27	203	191
Cu	23.75	14.48	22.7	26.0
Fe	23.29	17.11	38134	41941
K	17098	15907	10939	10580
Li	0.01	0.03	42.2	39.4
Mg	917	668	32099	39504
Mn	5.64	5.68	622	454
Na	300	644	7507	7448
Nb	0.03	0.07	5.55	5.74
Ni	1.43	1.09	262	248
P	8550	5395	224	215
Pb	0.29	0.32	36.0	30.8
Rb	480	356	140	115
S	9297	9339	695	691
Se	16.92	11.57	bdl	bdl
Sn	0.41	0.48	1.98	2.28
Sr	0.20	0.24	88.2	86.7
Ti	0.07	0.11	3482	3518
Zn	204	124	52.0	51.3
δ^26^ Mg ± 2SE	−0.60 ± 0.03	−0.57 ± 0.04	0.18 ± 0.03	0.29 ± 0.03
δ^65^Cu ± 2SE	−0.27 ± 0.02	−0.15 ± 0.01	0.81 ± 0.01	1.17 ± 0.02
δ^66^Zn ± 2SE	1.01 ± 0.01	0.88 ± 0.01	−0.04 ± 0.01	−0.14 ± 0.02
δ^114^Cd ± 2SE	−0.14 ± 0.02	−0.13 ± 0.02	0.50 ± 0.01	0.32 ± 0.01

bdl, below the detection limit

**Table 3:** Bioaccumulation (BF) and translocation (TF) factors for the studied mushroom sub-samples

BF				TF
	FB/soil	Stipe_bulk_/soil	Stipe_(0–3)_/soil	Stipe_(3–6)_/stipe_(0–3)_
Ag	3.15	5.98	1.47	2.55
Al	0.00	0.00	0.00	1.41
As	0.07	0.08	0.09	0.65
Ba	0.00	0.00	0.00	0.78
Ca	0.00	0.00	0.01	0.77
Cd	1.30	2.83	0.90	1.74
Cr	0.00	0.00	0.00	1.50
Cu	0.33	0.64	0.22	1.59
Fe	0.00	0.00	0.00	1.53
K	1.11	1.45	1.04	1.27
Li	0.00	0.00	0.00	0.42
Mg	0.01	0.02	0.01	1.24
Mn	0.01	0.01	0.01	0.98
Na	0.14	0.09	0.20	0.89
Nb	0.02	0.01	0.03	0.38
Ni	0.00	0.00	0.00	1.05
P	13.82	24.05	10.57	1.48
Pb	0.01	0.01	0.01	1.46
Rb	1.52	2.55	1.33	1.26
S	13.30	13.44	16.04	0.93
Se	N/A	N/A	N/A	1.20
Sn	0.24	0.24	0.20	1.06
Sr	0.00	0.00	0.00	0.77
Ti	0.00	0.00	0.00	1.06
Zn	1.18	2.38	0.56	2.35

TF				
	Stipe_(6–9)_/stipe_(3–6)_	Cap/stipe_(6–9)_	Cap/stipe_bulk_	Spor./cap
Ag	1.38	0.97	1.59	1.94
Al	1.38	1.49	2.28	0.45
As	1.80	0.72	1.08	1.23
Ba	0.80	0.79	0.87	0.70
Ca	0.67	1.14	0.95	0.79
Cd	1.17	1.19	1.67	2.29
Cr	1.18	0.99	1.53	1.59
Cu	1.42	1.07	1.65	1.95
Fe	1.49	0.93	1.41	1.41
K	1.19	1.12	1.58	0.89
Li	0.66	0.47	0.25	0.11
Mg	1.15	1.07	1.44	1.42
Mn	0.89	1.35	1.37	0.84
Na	0.74	0.52	0.48	0.58
Nb	2.44	0.38	0.64	0.39
Ni	1.17	0.91	1.22	1.42
P	1.26	1.00	1.44	1.92
Pb	0.68	0.88	0.81	1.00
Rb	1.28	1.29	1.82	1.24
S	0.92	1.00	1.03	0.98
Se	1.06	1.11	1.36	1.66
Sn	0.95	1.47	1.26	0.68
Sr	0.65	1.13	0.87	0.82
Ti	1.37	1.62	2.75	0.34
Zn	1.54	1.00	1.70	1.96

FB, fruiting body. Spor., sporophore, N/A, not applicable

Isotopic compositions of the studied mushroom sub-samples were characterized by the values of δ^26^Mg from −1.04 to −0.31‰, δ^66^Zn from + 0.69 to + 1.01‰, δ^65^Cu from -0.33 to + 0.30‰ and δ^114^Cd from −0.10 to −0.15‰. The mushroom preferentially took up lighter Mg, Cu and Cd isotopes (Δ^26^Mg_fruiting body-soil_ = -0.75‰; Δ^65^Cu_fruiting body-soil_ =  −0.96‰; Δ^114^Cd_fruiting body-soil_ = -0.63‰), and a heavier isotope of Zn (Δ^66^Zn_fruiting body-soil_ =  + 0.92‰).

No systematic differences between the elemental compositions of the two analyzed substrate sub-types were observed. Some elements displayed slightly higher concentrations in the mushroom-bearing substrate (e.g., Al, K, Mn, Pb, Rb) whereas others were present in higher amounts in the mushroom- “free” substrate (e.g., Cu, Fe, Mg, Ti). However, most elements did not show pronounced differences in concentrations between these two substrate sub-types.Unlike for elemental composition, some differences in isotopic compositions of the two analyzed substrate types were observed: δ^26^Mg values were + 0.18 and + 0.29‰, δ^66^Zn were -0.14 and -0.04‰, δ^65^Cu were + 0.81 and + 1.17‰, and δ^114^Cd were + 0.32 and + 0.50‰. Chemical and isotopic compositions of mushroom and substrate samples are given in Table 2.

## Discussion

### Elemental features

Elements displaying high BFs (bioaccumulating elements) translocated within the mushroom’s fruiting body more readily than elements with low BFs (bioexclusive elements). Therefore, selection of the “useful” elements for further within-mushroom translocation began immediately after the uptake. Since fungi may have very significant geochemical influence within the terrestrial environment (Gadd 2017), such selectivity is a component of biogeochemical cycles for metals and associated elements.

The constant decrease of the low mobile elements concentrations towards the upper parts of the mushroom’s fruiting body was observed: 0–3 cm > 3–6 cm > 6–9 cm > cap > sporophore (cap sometimes displayed higher concentrations than the sporophore; Table 2). The mobile elements translocated through the stipe and accumulated preferentially in the cap and sporophore: 0–3 cm < 3–6 cm < 6–9 cm < cap < sporophore (for otherwise mobile Al and K, the cap displayed higher concentrations). Arsenic and Mn translocated through the fruiting body unevenly, not following the above-described schemes. Arsenic displayed a pulse-like distribution, whereas Mn displayed steady decrease of the concentrations along the stipe and then accumulation in the cap.

Since no systematic differences were observed between the elemental compositions of the two analyzed soil sub-types, we cannot say with certainty yet how the mushroom’s uptake influenced the elemental composition of the substrate.

### Isotopic features

The interpretation of isotope fractionation in biological systems is not straight-forward because fractionation is due to lots of factors relating to different natural processes some of which could be unknown and not taken into the account. Overall, isotope fractionation in various biological systems takes place during uptake, transport and internal cycling of elements. Presently obtained data showed that isotopes of all four considered systems (Mg, Cu, Zn, and Cd) fractionated to various extent both at the substrate to mushroom interface and within the mushroom’s fruiting body.

#### Soil to mushroom interface (Mg)

**Fig. 2:**
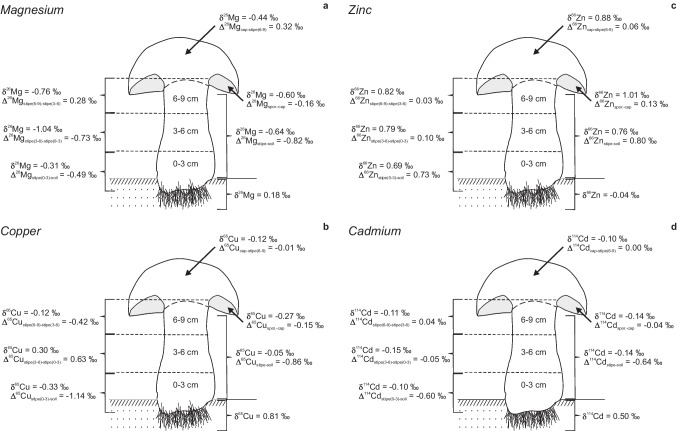
Isotopic characteristics of the studied sample of the *Boletus edulis* mushroom; a, Mg isotopes; b, Cu isotopes; c, Zn isotopes; d, Cd isotopes. Spor., sporophore. The studied sections of the mushroom’s stipe are depicted as 0–3 cm, 3–6 cm and 6–9 cm. See text for details

The current data on Mg isotopes for a *Boletus edulis* sample suggest that the mushroom preferentially took up the lighter ^24^ Mg isotope (Δ^26^Mg_stipe(0–3)-soil_ = -0.49‰; Δ^26^Mg_fruiting body-soil_ = -0.75‰) (Fig. 2a; Table 4). Generally, during various biochemical processes, lighter isotopes incorporate more favorable in products mobilized from substrates enriched in heavier isotopes (see Shearer and Kohl 1986; Hobbie and Högberg 2012). For example, the signatures of δ^26^Mg depletion were observed in cyanobacteria (Black et al. 2008) and in the laboratory grown ectomycorrhizal fungi (Fahad et al. 2016). In the case considered here, significant excess of Mg (3.2–4.0 wt.%) enriched in heavier isotope (Table 2) in serpentinite-based substrate may enable *B. edulis* to fractionate Mg negatively. It was suggested by Pokharel et al. (2018) and Wiggenhauser et al. (2022) that isotopically distinguishable Mg pools exist in vascular plants ‘ tissues. Whereas most plant ‘s tissues favor a light ionic Mg pool, a heavy Mg pool stores in chlorophyll responsible for accumulation of heavy Mg isotopes in certain tissues of plants. However, since mushrooms do not need chlorophyll for their life circle, formation of chlorophyll-bearing complexes cannot be responsible for within-mushroom isotope fractionation. It could be suggested then that isotopically light ionic Mg could be the major form of the element responsible for negative Mg fractionation at the soil to mushroom interface.

**Table 4:** Fractionation factors (Δ) for the studied mushroom sub-samples

	FB-soil	Stipebulk-soil	Stipe_(0–3)_-soil	Stipe_(3–6)_-stipe_(0–3)_	Stipe_(6–9)_-stipe_(3–6)_
Δ^26^ Mg	−0.75	−0.82	−0.49	−0.73	0.28
Δ^65^Cu	−0.96	−0.86	−1.14	0.63	−0.42
Δ^66^Zn	0.92	0.80	0.72	0.10	0.03
Δ^114^Cd	−0.63	−0.64	−0.60	−0.05	0.05

	Cap-stipe(6–9)	Cap-stipebulk	Spor.-cap
Δ^26^ Mg	0.32	0.20	−0.16
Δ^65^Cu	−0.01	−0.08	−0.15
Δ^66^Zn	0.06	0.12	0.13
Δ^114^Cd	0.00	0.04	−0.04

FB, fruiting body; Spor., sporophore

#### Soil to mushroom interface (Cu)

Analyses revealed preferential uptake of the lighter ^63^Cu isotope by the mushroom. For redox sensitive transition metals such as Cu, reduction can be required prior to the uptake, and significant light Cu isotopic enrichment would be due to the reduction of free Cu^2+^ to Cu^+^ (see Criss 1999; Zhu et al. 2002; Kavner et al. 2008; Navarette et al. 2011; Weinstein et al. 2011; Jouvin et al. 2012; Ryan et al. 2013; Li et al. 2016; Blotevogel et al. 2022; Wiggenhauser et al. 2022). Therefore, observed significant negative Cu isotope fractionation between the substrate and the *B. edulis* fruiting body (Δ^65^Cu_stipe(0–3)-soil_ =  −1.14‰; Δ^65^Cu_fruiting body-soil_ = -0.96‰) (Fig. 2b; Table 4) indicates a dominant reductive uptake mechanism. This is consistent with negative soil to plant fractionation reported for vascular plants (Weinstein et al. 2011; Ryan et al. 2013) and with the substrate to mushroom fractionation (Andronikov et al. 2022). Not only redox-induced, but also kinetic isotope fractionation could contribute to the enrichment of biological systems with the lighter ^63^Cu isotope (Hindshaw et al. 2013). Such fractionation may be due to the change of active Cu uptake to passive Cu uptake because of increased element availability (see Blotevogel et al. 2022; Wiggenhauser et al. 2022). This, however, unlikely occurs at low exposure to Cu, which was the case for the studied catchment (Table 2). Therefore, observed significant negative Cu isotope fractionation at the substrate to mushroom interface was mainly redox-induced equilibrium fractionation. That is to say, the redox state was supposed to change from the preferentially oxidizing to preferentially reducing at the substrate to mushroom interface.

#### Soil to mushroom interface (Zn)

Unlike in the cases of Mg and Cu, higher uptake of the heavier ^66^Zn isotope (Δ^66^Zn_stipe(0–3)-soil_ =  + 0.72‰; Δ^66^Zn_fruiting body-soil_ =  + 0.92‰) was demonstrated by the *B. edulis* mushroom (Fig. 2c; Table 4). Such behavior of Zn isotopes is similar to that observed for some vascular plants (Weiss et al. 2005; Viers et al. 2007; Moynier et al. 2009; Arnold et al. 2015; Kříbek et al. 2020), and for a sample of the *X. subtomentosus* mushroom collected from the amphibolite-based NAZ catchment (Andronikov et al. 2022). Since Zn is not a redox-sensitive transient metal (it has only one oxidation state of Zn^2+^), the redox reactions would not result in Zn isotope fractionation. Fungi may affect growing substrate both biomechanically (when hyphae grow through grain boundaries, cleavages and cracks) and biochemically (when fungi solubilize substrate’s minerals releasing metal compounds). We did not consider the biomechanical aspect of the mushroom-to-substrate interaction. Mycorrhizal fungi can dissolve a mineral component of soils and rocks primarily via acidolysis (Burford et al. 2003; Martino et al. 2003; Fomina et al. 2004; Gadd 2007) that results in mobilization of metals and other nutrients from minerals. Recent research showed that mushrooms may release organic acids (mostly, oxalic acid) which aggressively attack mineral surfaces and form metal–organic complexes (mostly oxalates) (Arnott 1995; Landweert et al. 2001; Gadd 2007; 2017; Gadd et al. 2014; Dusemgemungu et al. 2021). Most formed this way solids are water-soluble. We suggest that the organic acids (mostly oxalic acid) preferentially would form complexes with Zn enriched in the heavier ^66^Zn isotope. But this is just a suggestion which requires much more comprehensive studies including biochemical study.

#### Soil to mushroom interface (Cd)

Our study of Cd isotope systematics showed preferential uptake of the lighter ^110^Cd isotope with significant negative Cd substrate to mushroom fractionation (Δ^114^Cd_stipe(0–3)-soil_ =  −0.60‰; Δ^114^Cd_fruiting body-soil_ = -0.63‰) (Fig. 2d; Table 4). Such a scheme of fractionation is consistent with the data available for some vascular plants (Wei et al. 2016; Imseng et al. 2019; Zhou et al. 2020; Wiggenhauser et al. 2021), with the data on Cd isotopic behavior in the system substrate to *Thelephora penicillata* mushroom (Borovicka et al. 2022), and with observations that biochemical processes in general favor initial incorporation of the lighter isotopes in products mobilized from substrates (Shearer and Kohl 1986; Hobbie and Högberg 2012; Jouvin et al. 2012; Ryan et al. 2013; Fahad et al. 2016). The oxidation state of Cd at the environmental conditions is Cd^2+^, and Cd displays chemical properties similar to those of Zn^2+^ (Thornton 1986). As a consequence, Cd can replace Zn during uptake from the substrate. However, the observed behavior of Cd isotopes was opposite to what we saw in the case of Zn: the mushroom preferentially took up the heavier ^66^Zn isotope and the lighter ^110^Cd isotope from the substrate. The opposing isotope fractionation of Zn and Cd in biological systems is known, and was described by Wiggenhauser et al. (2021; 2022) and Zhao et al. (2021) for vascular plants. These authors suggested that the opposing isotope fractionation of the elements can be induced by dehydration and the difference in stability of the element’s hydration spheres. The isotope shift toward lighter ^110^Cd isotopes was suggested to be due to dehydration of Cd from Cd(H_2_O)_6_ to Cd(H_2_O)_5_.

#### Within-mushroom isotope fractionation of Mg

Metals can be transported within fungal cells by different means and, in particular, by metal-binding proteins (e.g., Benes et al. 2019; Sacky et al. 2022). Individual metal transporters may prefer either lighter or heavier isotopes that results in isotope fractionation. Our study showed that a degree of within-mushroom isotope fractionation is element-dependent. The analyzed stipe subsamples (0–3 cm, 3–6 cm, and 6–9 cm), and cap and sporophore samples (Fig. 2) displayed different Mg isotopic compositions. That was especially pronounced for a middle (3–6 cm) stipe section. This section displayed significant depletion in the heavier ^26^ Mg isotope with respect to both a lower (Δ^26^Mg_stipe(3–6)-stipe(0–3)_ =  −0.73‰) and an upper (Δ^26^Mg_stipe(6–9)-stipe(3–6)_ =  + 0.28‰) stipe sections (Fig. 2a; Table 4). Therefore, Mg isotopes did not fractionate steadily along the mushroom’s fruiting body, but formed isotopically distinct Mg pools. Further translocation of Mg to upper parts of the mushroom’s fruiting body led to enrichment of the cap in the heavier ^26^ Mg isotope with respect to both the 6–9 cm stipe section (Δ^26^Mg_cap-stipe(6–9)_ =  + 0.32‰) and the sporophore (Δ^26^Mg_sporophore-cap_ =  −0.16‰). Overall enrichment of the cap in the heavier ^26^ Mg isotope compared to the mushroom’s bulk stipe (Δ^26^Mg_cap-stipe_ =  + 0.20‰) suggests preferential upward translocation of the heavier ^26^ Mg isotope.

#### Within-mushroom isotope fractionation of Cu

Similar to Mg, copper displayed uneven within-mushroom isotope fractionation scheme with isotopically distinct pools available. The stipe section of 3–6 cm was strongly enriched in the heavier ^65^Cu isotope with respect to the 0–3 cm (Δ^65^Cu_stipe(3–6)-stipe(0–3)_ =  + 0.63‰) and 6–9 cm (Δ^65^Cu_stipe(6–9)-stipe(3–6)_ = -0.42‰) sections (Fig. 2b; Table 4). That is, a significant portion of the heavier ^65^Cu isotope accumulated in the middle section of the stipe (3–6 cm). In spite of this, no specific features were observed for the elemental Cu distribution and behavior: copper as well as other mobile elements (preferentially accumulated in the upper parts of the mushroom’s fruiting body (5.0 mg kg^−1^ Cu in the lowermost part of the stipe vs. 24 mg kg^−1^ Cu in the sporophore). Virtually no Cu isotope fractionation was observed between the uppermost part of the stipe (6–9 cm) and the cap (Δ^65^Cu_cap-stipe(6–9)_ =  −0.01‰). The sporophore slightly fractionated Cu negatively with respect to the cap (Δ^65^Cu_sporophore-cap_ =  −0.15‰). The observed scheme of the within-mushroom Cu isotope fractionation suggests that different parts of the mushroom accumulated distinct Cu isotopes forming isotopically distinct pools (Fig. 2b). Unlike in the case of strong redox-related negative Cu isotope fractionation, insignificant enrichment in the light ^63^Cu isotope was ascribed to Cu immobilization by Cu^+^ complexation to S that favors light isotopes (Cadiou et al. 2017; Blotevogel et al. 2019). Some research also showed that the kinetic isotope fractionation could take place while Cu^+^ carried by the copper transporting proteins (CTR) passes across the plasma membrane (Dumay et al. 2006; Cadiou et al. 2017 and references therein). This would lead to the enrichment in the lighter ^63^Cu isotope. But this enrichment is not as strong as that in the case of the redox-induced fractionation. Positive Cu isotope fractionations between different parts of the mushroom do not seem to fit into a broader picture of Cu isotope cycling in natural systems where the reduction of Cu^2+^ to Cu^+^ controls the distributions of Cu isotopes and leads to significant negative Cu isotope fractionation. However, Li et al. (2016) and Wiggenhauser et al. (2022) suggested that reoxidation of Cu^+^ to Cu^2+^ could lead to the heavy ^65^Cu isotope enrichment. Therefore, it is possible that the redox reactions along with kinetic processes and Cu^+^ complexation to S could eventually lead to both positive (reoxidation) and negative (reduction) Cu fractionation.

#### Within-mushroom isotope fractionation of Zn

Such fractionation was characterized by insignificant though but measurable preferential translocation of the heavier ^66^Zn isotope upwards (Δ^66^Zn_cap-stipe(0–3)_ = 0.19‰; Δ^66^Zn_sporphore-stipe(0–3)_ = 0.32‰) (Fig. 2c). Unlike Mg and Cu, Zn did not form isotopically different pools in the mushroom’s fruiting body. Within-stipe Zn isotope fractionation was very insignificant (Δ^66^Zn_stipe(3–6)-stipe(0–3)_ =  + 0.10‰; Δ^66^Zn_sstipe(6–9)-stipe(3–6)_ =  + 0.03‰) (Fig. 2c; Table 4). Similarly, only very insignificant fractionation was observed between the uppermost part of the stipe (6–9 cm) and the cap (Δ^66^Zn_cap-stipe(6–9)_ =  + 0.06‰). The positive Zn fractionation between the sporophore and the cap (Δ^66^Zn_sporophore-cap_ =  + 0.13‰) was similar to that reported by Andronikov et al. (2022) for a sample of *X. subtomentosus* from the amphibolite-based substrate (Δ^66^Zn_sporophore-cap_ =  + 0.09‰). Steady positive upward within-mushroom Zn isotope fractionation could be explained by a successive precipitation of lighter isotopes on the hyphea walls or on septa. Such precipitation likely led to the enrichment of heavier isotopes in the soluble Zn fraction which was then further transported by the transpiration flow. In the end points of the transpiration flow such as the mushroom’s cap and sporophore, only a small fraction of the total Zn was stored but this fraction was most enriched in the heavier ^66^Zn isotope (Δ^66^Zn_sporophore-stipe(0–3)_ =  + 0.32‰).

#### Within-mushroom isotope fractionation of Cd

 It was very insignificant compared to other considered isotope systems (Fig. 2d; Table 4). Measurable within-mushroom Cd isotope fractionation was observed for the stipe. The stipe section 3–6 cm was slightly depleted in the heavier ^114^Cd isotope with respect to the 0–3 cm (Δ^114^Cd_stipe(3–6)-stipe(0–3)_ =  −0.05‰) and 6–9 cm (Δ^114^Cd_stipe(6–9)-stipe(3–6)_ =  + 0.05‰) sections. No Cd isotope fractionation occurred between the uppermost part of the stipe (6–9 cm) and the cap (Δ^114^Cd_cap-stipe(6–9)_ =  + 0.00‰). The sporophore fractionated Cd isotopes very insignificantly with respect to the cap (Δ^114^Cd_sporophore-cap_ = -0.04‰). The observed features suggest that virtually no within-mushroom Cd isotope fractionation occurred for the *B. edulis* sample (Δ^114^Cd values were only slightly above the analytical errors). Although Cd displayed a tendency to accumulate the heavier ^114^Cd isotope in the upper parts of the mushroom’s fruiting body, this tendency was very subtle (Δ^114^Cd_cap-stipe_ =  + 0.04‰). Such insignificant Cd isotope fractionation can be best explained by fungal-driven fractionation processes. Overall subtle (if at all) within-mushroom Cd isotope fractionation differs from much more significant isotope fractionation reported for vascular plants (Wiggenhauser et al. 2021; 2022). However, unlike in plants, the information about the molecular mechanisms involved in within-mushroom Cd translocation is missing. Overall, the only reliably measured Cd isotope fractionation occurred at the substrate to mushroom interface (Δ^114^Cd_fruiting body-soil_ =  −0.63‰). This number is close to that reported by Borovicka et al. (2022) at the interface of the soil to the *Thelephora pinicellium* mushroom (Δ^114^Cd_fruiting body-soil_ =  −0.41‰).

#### Unusual behavior of Mg and Cu isotopes

Within-mushroom behavior of isotopes of these two systems was not as straight-forward as one could expect. Instead of a steady fractionation (like Zn) or virtually no fractionation (like Cd), the mushroom’s stipe displayed the presence of isotopically distinct pools of both Mg and Cu (Fig. 2a, b). As of now, we cannot say with certainty why these two isotope systems behaved the way observed. Although isotopically distinguishable Mg pools were reported to exist in vascular plants (Pokharel et al. 2018; Wiggenhauser et al. 2022), there are no obvious reasons for the presence of such pools in the studied mushroom sample. We can exclude influence of the selective contamination because all mushroom sub-samples were prepared simultaneously by the same way, and were cleaned of all debris (mineral and organic) very carefully.

Overall, mushrooms have a very simple structure and are composed of apically growing hyphae which usually have a diameter of 1–30 μm or more, depending on fungal species and growth conditions (Walker and White 2018). The hyphae translocates (and apparently fractionates) available elements. Although we did not study the structure of the stipe of the *B. edulis* sample in detail, the lower part of the stipe was much denser than the middle and upper parts. This can lead to decrease of the diameter of individual hypha and to increase of the pressure of nutrient/mineral-bearing solutions on the hypha walls. Magnesium can be taken by mushrooms from the substrate as ionic Mg^2+^ and as Mg in organic-metal complexes (Walker and White 2018). The ionic Mg^2+^ is favoring light isotopes, whereas Mg in the organic-metal complexes is favoring heavy isotopes (Bolou-Bi et al. 2010). We can suggest then that Mg in the organic-metal complexes preferentially precipitates on the hypae walls and on septa in the denser (lower) part of the stipe, whereas ionic Mg^2+^ would continue to transport to the upper parts of the mushroom’s fruiting body. That could explain significant negative Mg fractionation in the stipe section 3–6 cm relative to the denser (lower) section of 0–3 cm. Further within-mushroom Mg fractionation would follow a positive trend (Fig. 2a) also observed for vascular plants and for a sample of *X. subtomentosus* mushroom from the amphibolite-based catchment (Bolou-Bi et al. 2012; Novak et al. 2020; Andronikov et al. 2022).

Copper is mostly taken from substrates as Cu^+^ formed by the reduction of Cu^2+^. This leads to significant negative Cu isotope fractionation (Weinstein et al. 2011; Ryan et al. 2013; Wiggenhauser et al. 2022). On the other hand, reoxidation of Cu^+^ to Cu^2+^ may result in enrichment of the heavier ^65^Cu isotope (Wiggenhauser et al. 2022). We suppose, that reoxidation could explain unsusual behavior of Cu isotopes, i.e., the redox state should somehow change along the length of the stipe resulting in formation of isotopically distinct Cu pools. Further insignificant negative Cu isotope fractionation in the fruiting body upwards (Δ^65^Cu_cap-stipe(6–9)_ =  −0.01‰; Δ^65^Cu_sporophore-cap_ =  −0.15‰) could be due to Cu complexation to S (Cu^+^-S) that favors the lighter ^63^Cu isotope (see Cadiou et al. 2017; Blotevogel et al. 2019) and/or kinetic fractionation during the passage of Cu^+^ carried by the CTR across the plasma membrane (Dumay et al. 2006; Cadiou et al. 2017). Although proposed isotope fractionation scenario could explain unusual within-mushroom behavior of Mg and Cu isotopes, it is more likely that the isotope fractionation scheme observed in the stipe of the *B. edulis* sample was related to lots of factors, among which the cellular structure and cell membrane permeability would be dominating. However, we would step into the realm of biochemistry and physiology here, which are we not the experts in. Anyway, it would be very important for further studies to analyze Cu speciation and the redox potential (Eh) in different mushroom’s parts and in the substrate.

#### Isotopic features of the substrate

Mushroom-bearing substrate displayed enrichment in lighter isotopes compared to the mushroom- “free” substrate for two out of four considered isotope systems (Mg and Cu). Such behavior of the isotopes seems unusual because the mushroom preferentially took up lighter isotopes of Mg and Cu from the substrate, and we would expect depletion of the mushroom-bearing substrate in lighter Mg and Cu isotopes. We can propose two explanations for the observed inconsistencies. The first one would suggest that the mushroom took up only very insignificant amounts of Mg and Cu from the substrate (both elements are bioexclusive) compared to the total amount of the elements there. These small amounts would not affect either elemental or isotopic compositions of the substrate up to the measurable levels. Observed behavior of Cd isotopes would be in favor of such a suggestion. Mushroom-bearing substrate was measurably enriched in the heavier ^114^Cd isotope (δ^114^Cd = 0.50‰) compared to the mushroom- “free” substrate (δ^114^Cd = 0.32‰). This is consistent with the preferential uptake of lighter Cd isotopes by the mushroom. Since the studied substrate samples were very poor in Cd (0.59–0.77 ppm), even an insignificant amount of Cd taken up by the mushroom could influence both Cd elemental and isotopic compositions of the substrate. However, such explanation would not explain observed behavior of Zn isotopes. Since concentrations of Zn in the substrate studied were low (51–52 ppm), and Zn is rather a bioaccumulating element, we would expect that uptake of the heavier ^66^Zn isotope could change isotopic composition of the substrate toward enrichment in the lighter ^64^Zn isotope. However, on the contrary, the mushroom-bearing substrate displayed enrichment in the heavier ^66^Zn isotope compared to the mushroom- “free” substrate (Table 2). Therefore, we propose the second explanation for the “isotopic inconsistencies” observed between the mushroom-bearing and mushroom- “free” substrates. This explanation would suggest that the lateral inhomogeneity of the substrate (1.5 m between the mushroom-bearing and mushroom- “free” varieties) was much more significant than any elemental and/or isotopic differences induced by mushroom’s uptake. Behavior of Cd isotopes would neither contradict nor support such a suggestion.

## Conclusions

A *Boletus edulis* mushroom studied has behaved as an accumulating biosystem for elements such as P, S, K, Zn, Se (which are bioessential elements), Ag, Cd and Rb, with other analyzed elements being bioexclusive. Within-mushroom distribution of elements was characterized by low TF values being mostly around 1.0 to 2.0, but with the extremes being as low as 0.3 for Ti, and as high as 2.6 for Ag. Bioaccumulating (BF > 1) elements translocated through the mushroom’s fruiting body more readily (accumulating preferably in the upper parts of the fruiting body) than bioexclusive (BF < 1) elements (remaining preferentially in the stipe).

A studied sample of the *B. edulis* was characterized by higher uptake of heavier isotopes of Zn, and lighter isotopes of Mg, Cu, and Cd. Only Zn displayed steady positive within-mushroom isotope fractionation. Cadmium did not show any significant within-mushroom isotope fractionation. Isotopes of Mg and Cu fractionated unevenly forming isotopically distinct pools in the mushroom’s fruiting body. A middle part of the stipe (3–6 cm) behaved unusually in terms of the Mg and Cu isotopes. It was strongly depleted in the heavier ^26^ Mg isotope with respect to both the 0–3 cm and 6–9 cm stipe sections. The 3–6 cm stipe section displayed strong enrichment in the heavier ^65^Cu isotope with respect to both the 0–3 cm and 6–9 cm sections. In the case of Mg, depletion in the heavier ^26^ Mg isotope could be due to different degrees of translocation of the ionic Mg^2+^ (favoring light isotopes) and Mg in organic-metal complexes (favoring heavy isotopes). Presence of isotopically different Cu pools can be due to the changing redox conditions within the mushroom’s fruiting body.

Substrate affected by interaction with the mushroom’s mycelium was richer in lighter Mg, Cu and Cd isotopes, and in the heavier ^66^Zn isotope than the substrate unaffected by such interaction. It could be due to the following: (i) mushroom took up only very insignificant amounts of Mg and Cu which amounts would not affect either elemental or isotopic compositions of the substrate; (ii) the lateral substrate heterogeneity was more significant than any elemental and/or isotopic differences induced by the uptake. More detailed mushroom-bearing *vs*. mushroom-free substrate studies are necessary in order to estimate influence of the mushroom’s uptake on elemental and isotopic compositions of the substrate.

## Data Availability

The datasets used and analyzed during the current study are available from the corresponding author on reasonable request.
